# ﻿Molecular data from the holotype of the enigmatic Bornean Black Shrew, *Suncusater* Medway, 1965 (Soricidae, Crocidurinae), place it in the genus *Palawanosorex*

**DOI:** 10.3897/zookeys.1137.94217

**Published:** 2022-12-21

**Authors:** Jonathan A. Nations, Thomas C. Giarla, Muhd Amsyari Morni, Julius William Dee, Mark T. Swanson, Anna E. Hiller, Faisal Ali Anwarali Khan, Jacob A. Esselstyn

**Affiliations:** 1 Museum of Natural Science, Louisiana State University, Baton Rouge, LA 70803, USA; 2 Department of Biological Sciences, Louisiana State University, Baton Rouge, LA 70803, USA; 3 Current Address: Department of the Geophysical Sciences, University of Chicago, Chicago, IL 60637, USA; 4 Current Address: Field Museum of Natural History, Chicago, IL 60605, USA; 5 Department of Biology, Siena College, 515 Loudon Rd., Loudonville, NY 12211, USA; 6 Faculty of Resource Science and Technology, Universiti Malaysia Sarawak, 94300 Kota Samarahan, Sarawak, Malaysia

**Keywords:** Borneo, *Palawanosorex*, Southeast Asia, Sunda Shelf, ultraconserved elements

## Abstract

Although Borneo has received more attention from biologists than most other islands in the Malay Archipelago, many questions regarding the systematic relationships of Bornean mammals remain. Using next-generation sequencing technology, we obtained mitochondrial DNA sequences from the holotype of *Suncusater*, the only known specimen of this shrew. Several shrews collected recently in Sarawak are closely aligned, both morphologically and mitochondrially, with the holotype of *S.ater*. Phylogenetic analyses of mitochondrial sequences indicate that the *S.ater* holotype and new Sarawak specimens do not belong to the genus *Suncus*, but instead are most closely related to *Palawanosorexmuscorum*. Until now *Palawanosorex* has been known only from the neighboring Philippine island of Palawan. Additional sequences from nuclear ultra-conserved elements from the new Sarawak specimens strongly support a sister relationship to *P.muscorum*. We therefore transfer *ater* to *Palawanosorex*. The new specimens demonstrate that *P.ater* is more widespread in northern Borneo than previously recorded. Continued sampling of Bornean mammal diversity and reexamination of type material are critical in understanding the evolutionary history of the biologically rich Malay Archipelago.

## ﻿Introduction

The biological richness of Borneo inspired the fields of evolutionary biology and biogeography (Wallace 1869). Nevertheless, Borneo’s flora and fauna remain woefully understudied. One mammalian group that exemplifies this problem is the white-toothed shrews (Soricidae, Crocidurinae). Currently, five species are recognized from the island, three in the genus *Crocidura* Wagler, 1832 (*C.foetida* Peters, 1870, *C.neglecta* Jentkin,1888, and *C.baluensis* Thomas, 1898) and two in the genus *Suncus* Ehrenberg, 1832 (*S.ater* Medway, 1965, and *S.hosei* Thomas, 1893). However, uncertainty remains regarding the number of species, particularly due to the presence of three named subspecies of *C.foetida* and the possible presence of *C.nigripes* Miller & Hollister, 1921 (Hinckley et al. 2022). The lack of clarity regarding the diversity of shrews from Borneo is primarily due to the paucity of specimens from the island and, secondarily, a lack of genetic data from type material.

Arguably the most enigmatic shrew from Borneo is the Black Shrew, *Suncusater*, which, to our knowledge, is known only from the holotype. The holotype (MCZ 36547; Museum of Comparative Zoology, Harvard University, Cambridge, MA, USA) was collected in 1937 around 1675 m (5,500 ft) elevation on Mount Kinabalu, Sabah, Malaysia (Griswold 1939). It was originally identified as *C.foetida*. However, Medway (1965) revisited the shrews of Borneo and determined that this specimen represented an undescribed species of the widespread genus *Suncus*. The generic identification was largely attributed to the presence of a fifth unicuspid that is characteristic of *Suncus* but is lacking in *Crocidura*. Additionally, the dark black pelage, dark hands and feet, and short tail relative to head-body length clearly distinguished the specimen from *C.foetida*. Medway (1965) suggested that this shrew is vastly different from any other Southeast Asian shrew but closely aligned with *Suncusdayi* Dobson, 1888 from southern India.

**Figure 1.**
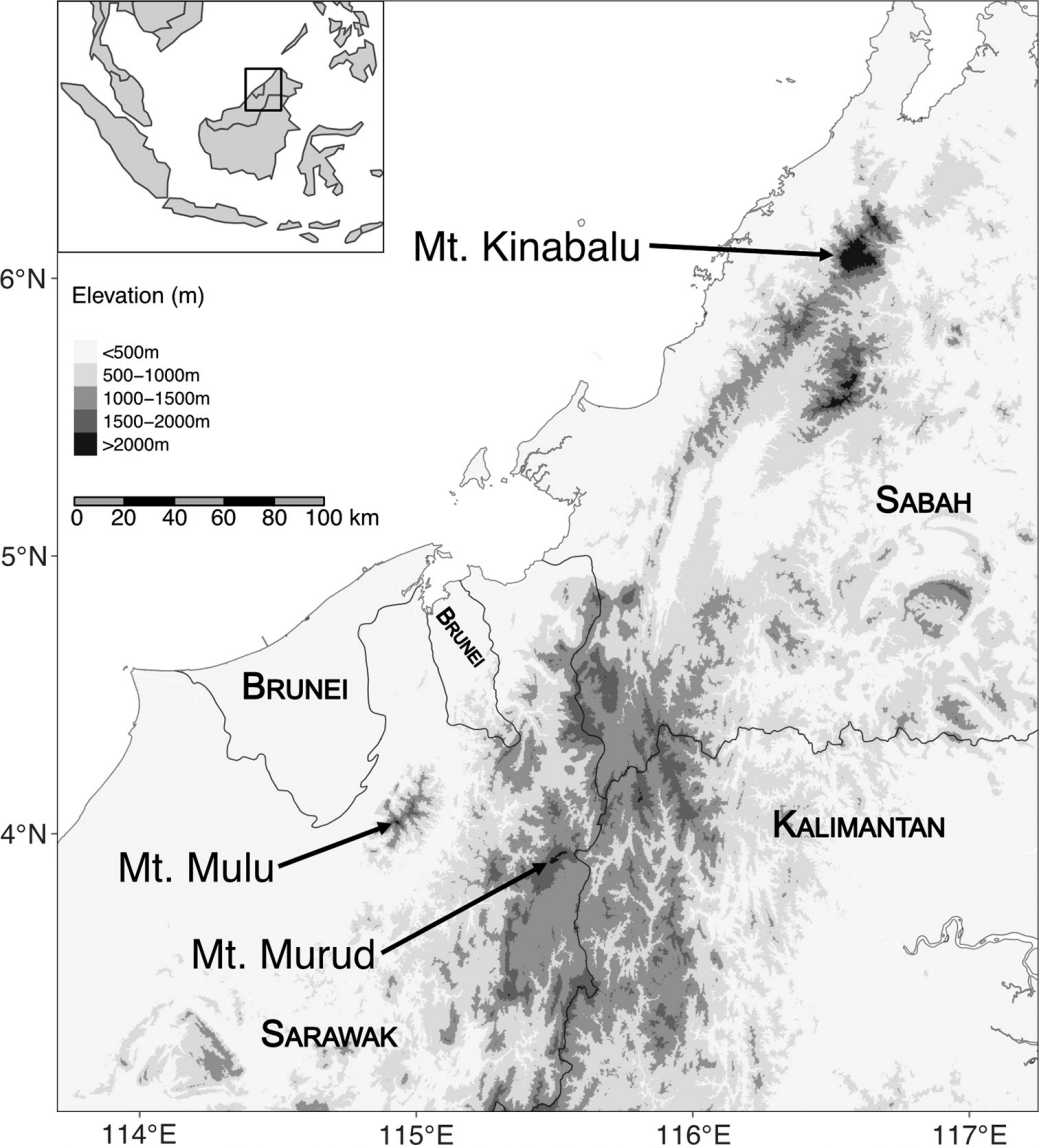
Map of northern Borneo showing the type locality of *Suncusater* (Mount Kinabalu) and recently surveyed sites in Sarawak (Mounts Mulu and Murud).

No other specimens of *S.ater* have been reported in the literature. However, a single specimen labeled as *S.ater* is cataloged in the Field Museum of Natural History, Chicago, USA (FMNH 159012). We inspected this specimen and quickly determined it to be much smaller than the type of *S.ater* (length of skull = 14 mm vs 21 mm in the *S.ater* holotype), and instead it likely represents *S.hosei*, a putative member of the *Suncusetruscus* Savi, 1822 species complex (Corbet and Hill 1992; Hutterer 2005; Omar et al. 2013). We recently sampled small mammals from two locations in northern Sarawak, Malaysia (Fig. 1) and recovered several medium-sized, dark-colored shrews with relatively short tails and a fifth unicuspid that match the physical description of *S.ater* (Medway 1965). We sequenced mitochondrial DNA from the holotype of *S.ater* and mitochondrial and nuclear DNA from the new Sarawak specimens to determine the phylogenetic placement of the holotype and our new specimens.

## ﻿Methods

### ﻿Fieldwork

We surveyed small mammals in two locations in Sarawak, Malaysia (Fig. 1): Mount Mulu (in 2017) and Mount Murud (in 2019). Both surveys used pitfall lines, which typically consisted of 5–10 large, 20–30 L buckets with a tarp drift fence, though occasionally we added smaller 1–3 L buckets. On Mount Mulu we set two pitfall lines of large buckets at 1650 m and one pitfall line of large buckets at 1800 m for a total of ca. 300 pitfall-nights. On Mount Murud we set pitfall lines (mixed large and small buckets) at 1480 m, 1660 m, 1770 m, 2000 m, 2250 m, and 2400 m for a total of ca. 440 pitfall-nights. Specimens were deposited at the Louisiana State University Museum of Natural Science, Baton Rouge, USA (LSUMZ). Specimens were measured, weighed, and then preserved in formalin (often with the skull removed and cleaned) or prepared as dried museum skins with dried and cleaned skeletons. Tissues were preserved in 95% ethanol. All collecting methods followed the recommended guidelines of the American Society of Mammalogists (Sikes et al. 2016).

### ﻿Specimen sampling, DNA extraction, and sequencing

Using a phenol-chloroform extraction protocol (Tsai et al. 2020), we extracted DNA from skin clips of the holotype of *S.ater* and a species from an outgroup genus (*Solisorexpearsoni* Thomas, 1924), as well as fresh tissues from three recently collected specimens from Sarawak that are morphologically aligned with *S.ater* (LSUMZ 40511, 40514, 40522). We modified the phenol-chloroform extraction of Tsai et al. (2020) by using a refrigerated centrifuge set at 3 °C during the ethanol precipitation steps. In addition to the phenol-chloroform extraction, we extracted genomic DNA from tissue samples (liver) from six Sarawak shrew specimens morphologically similar to *S.ater* (LSUMZ 40511, 40514, 40522, 40695, 40696, 40697) using ﻿Qiagen DNEasy Blood & Tissue kits (Qiagen, Germantown, Maryland) following the manufacturer’s instructions.

Phenol-chloroform extractions from skin clip samples (*S.ater* holotype and *S.pearsoni*) were treated with a New England BioLabs (Ipswich, MA) PreCR Repair Kit following the manufacturer’s instructions to repair preservation-related damage. For the phenol-chloroform extractions from the three fresh samples, we mechanically sheared the DNA to a 400–600 bp size range using an Epigentek Episonic sonicator. We prepared genomic libraries for all five phenol-chloroform extractions with a KAPA Hyper Prep kit and dual indexed iTru adapters (Glenn et al. 2019) following Esselstyn et al. (2017). Because the *S.ater* holotype extraction had a very small amount of DNA, we used diluted index primers at 1.25 μM (a quarter of the standard molarity) for this specimen. We then pooled the libraries and enriched them for the standard Tetrapods 5k loci (Faircloth et al. 2012) and 27 exons using the probe set introduced by Esselstyn et al. (2021) and manufactured by Arbor Biosciences (Ann Arbor, MI). We removed short fragments less than 150 base pairs from the enriched pools with a QIAGEN GeneRead Size Selection kit and confirmed the absence of adapter dimers with an Agilent Bioanalyzer using a DNA-High Sensitivity Kit. We then combined the enriched libraries into an equimolar pool with an unenriched library from the *S.ater* holotype specimen in order to enhance the likelihood of sequencing mitochondrial fragments from this specimen. Novogene (Beijing, China) sequenced these libraries on an Illumina HiSeq 4000 PE 150 lane (Illumina Inc., San Diego, CA, USA). For the six fresh tissue Qiagen DNEasy extractions, we amplified the mitochondrial protein coding gene cytochrome *b* [CYTB] using Polymerase Chain Reaction (PCR) following the protocol described in Esselstyn et al. (2009).

### ﻿Bioinformatics

We processed the UCEs in PHYLUCE v. 1.7.1 (Faircloth 2016), following Tutorial III guidelines. We processed raw Illumina reads with illumiprocessor (Faircloth 2013) and assembled trimmed reads into contigs using both Trinity v. r2013.08.14 (Grabherr et al. 2011) and SPAdes v. 3.14.1 (Prjibelski et al. 2020); we chose the assembler run that resulted in the largest number of UCE loci for final analyses. Contigs matching the UCE probes were aligned in MAFFT v. 7.475 (Katoh and Standley 2013) and edge-trimmed in PHYLUCE using trimmomatic (Bolger et al. 2014). Unfortunately, we did not obtain UCEs from the *S.ater* holotype. To obtain mitochondrial bycatch from the UCE data for three samples (the *S.ater* holotype, LSUMZ 40514, 40522) we also assembled reads into contigs using both metaSPAdes v. 3.14.1 (Nurk et al. 2017) and MEGAHIT (Li et al. 2016) as part of the MitoFinder pipeline (Allio et al. 2020). We then searched through the assembled contigs using several different mitochondrial genomes as references to improve our recovery of the mitochondrial data: *Suncusmurinus* Linnaeus, 1766, *Crociduraattenuate* Milne-Edwards, 1872, *C.dongyangjiangensis* Liu Yang et al., 2020, *C.lasiura* Dobson, 1890, *C.russula* Hermann, 1780, *C.grayi* (Dobson, 1890), *C.fuliginosa* Blyth, 1855, and *C.beata* Miller, 1910 (GenBank: NC_024604.1, KP120863.2, NC_056167.1, KR007669.1, NC_056768.1, KR537885.1, C_042762.1, KR537889.1). The resulting contigs were combined with the original Trinity or SPAdes contigs from the UCE assemblies and then aligned and annotated for 15 mitochondrial genes using the *Crocidurashantungensis* Miller, 1901 mitochondrial genome (GenBank: OM038325) as a reference in Geneious. Finally, we downloaded previously published mitochondrial and UCE sequences from other outgroups, mostly matching the taxon sampling in Hutterer et al. (2018).

We assembled an alignment of ten mitochondrial genes using a combination of newly generated sequences and sequences from GenBank (Suppl. material 1). For several outgroup species (*Crocidurapalawanensis* Taylor, 1934, *C.russula*, *C.sibirica* Dukelski, 1930, and *Suncusmurinus*), gene sequences were pulled from whole mitochondrial genomes that were available via GenBank. We included CYTB sequences from the recently collected Bornean material, as well as CYTB and 16s rRNA sequences from *Suncusdayi* to test the relationship proposed by Medway (1965). We partitioned the dataset by gene and, for all but 16s rRNA, by codon position (28 data subsets). We tested for the best partitioning scheme and best fitting model using ModelFinder (Kalyaanamoorthy et al. 2017) in IQ-TREE (Minh et al. 2020). We then conducted a maximum-likelihood (ML) phylogenetic analysis in IQ-TREE, assessing nodal support with 1000 ultrafast bootstrap replicates. In order to verify our results with a lower percentage of missing data, we constructed a second alignment of nine mitochondrial genes by removing eight samples that were only represented with CYTB. We repeated the same IQ-TREE analysis on this second mitochondrial DNA alignment of 20 samples. We generated CYTB*p*-distances for each sample in the first 28 specimen alignment, and the *p*-distances of nine mitochondrial genes from the second 20 specimen alignment.

We analyzed the 19-specimen UCE dataset using two different approaches: (1) a concatenated ML analysis, and (2) a two-step species tree analysis. For the concatenated analysis, we first identified a set of alignments that met two requirements: each had to comprise at least 14 sequences (i.e., 75% complete) and be at least 300 bp long. We concatenated the alignments in PHYLUCE and analyzed the dataset in IQ-TREE following the same protocol as for the mitochondrial dataset (except we did not partition the dataset). For the two-step species-tree analysis, we first inferred a gene tree in IQ-TREE for each UCE alignment with at least four sequences and at least 300 bp. For each alignment, we tested substitution models but did not estimate nodal support. We then subjected the resulting gene trees to TreeShrink (Mai and Mirarab 2018) to remove any outlier long branches. This final set of gene trees was then used as input for a species tree analysis in ASTRAL v. 5.7.7 (Zhang et al. 2018). Nodal support was measured using local posterior probabilities, a quartet-based support metric.

## ﻿Results

### ﻿Fieldwork

The 2017 and 2019 fieldwork in Sarawak recovered three species of shrews: *Crocidurafoetida*, *C.neglecta*, and several specimens of a medium-sized, dark-colored shrew with relatively short tails. Nearly all shrews were captured in pitfall traps. *Crocidurafoetida* and *C.neglecta* were captured in the same traplines as the dark-colored shrews, suggesting syntopy among all three species.

### ﻿Phylogenetics

**Figure 2.**
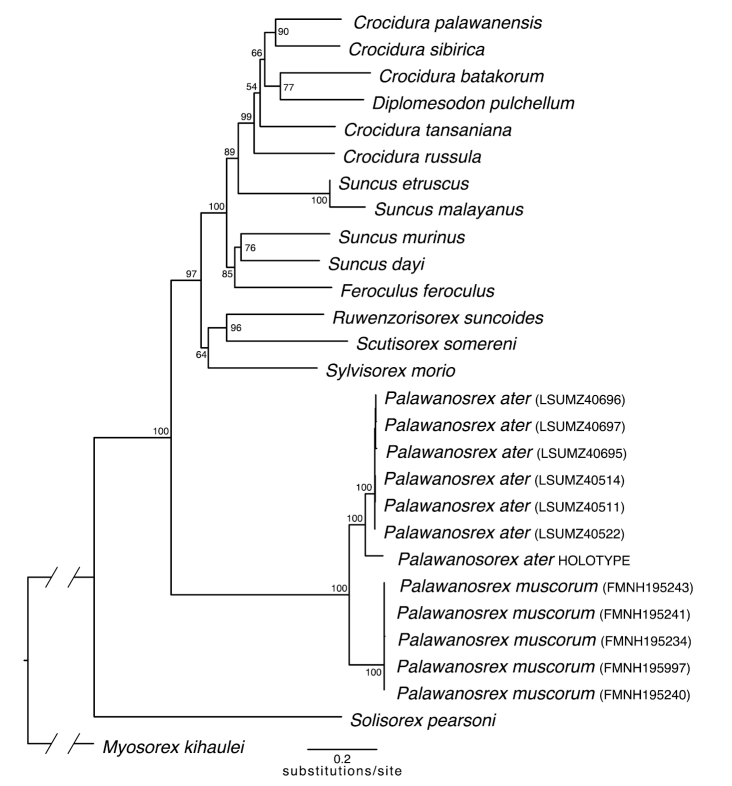
Maximum-likelihood crocidurine mitochondrial gene tree inferred in IQTree. Bootstrap values are given at the nodes. The holotype of *ater* (MCZ36574) forms a clade with the specimens recently collected in Sarawak. The outgroup branch to *Myosorexkihaulei* has been truncated.

The 10-gene mitochondrial dataset comprised 10,399 bp of sequence data; 48.0% of the data matrix was missing. Newly generated mitochondrial sequence data are available on GenBank (Suppl. material 1). ModelFinder partitioned the dataset into seven subsets and identified the best-fitting nucleotide substitution model for each (Suppl. material 2). The mitochondrial phylogeny shows that the holotype of *Suncusater* is sister to the six recently collected, dark-colored shrews from northern Sarawak (Fig. 2). Furthermore, the *S.ater* clade is sister to a clade of the Palawan endemic, *Palawanosorexmuscorum*Hutterer et al., 2018, a recently described crocidurine genus (Hutterer et al. 2018). The second nine-gene mitochondrial dataset had 29% missing data. The resulting estimated phylogeny is similar to the 10-gene topology, with the only changes occurring in the branching pattern of distantly related *Crocidura* taxa (Suppl. material 3). We found that the average CYTB*p*-distance between the five *P.muscorum* samples and the six newly collected, dark colored shrews is 11.77% (SD = 0.50%). These two species are distant relatives of species currently placed in the genus *Suncus*. The average mtDNA *p*-distance of *P.muscorum* and the two newly collected, dark-colored shrews in the nine-gene alignment, without CYTB, is 17.75%. The average nine-gene mtDNA *p*-distance of the *S.ater* holotype and the two newly collected, dark-colored shrews is 3.6%.

**Figure 3.**
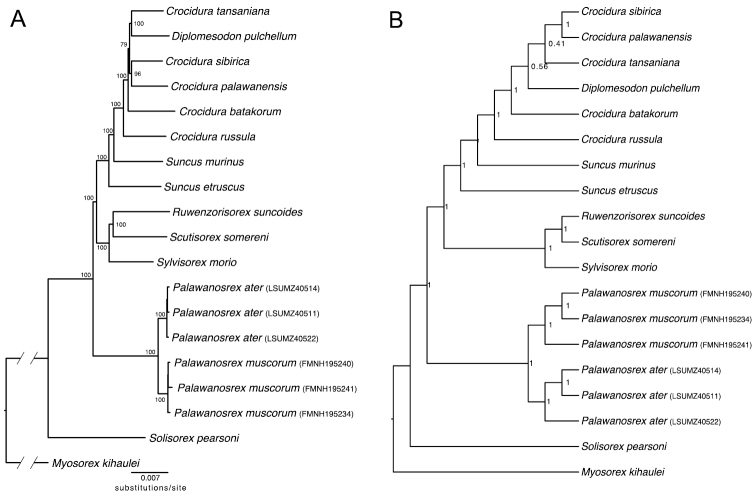
Phylogenetic hypotheses from UCE data. The tree topologies are very similar between the two methods. **A** phylogenetic tree inferred using 3,757 concatenated UCE loci (2,175,243 base pairs) in IQTree. Bootstrap supports are provided at the nodes. The branch leading to outgroup *Myosorexkihaulei* has been truncated for display **B** species tree inferred using ASTRAL. Nodal supports are given in local posterior probabilities. ASTRAL tree is presented as ultrametric with uninformative branch lengths.

The complete UCE dataset (which included only those alignments with more than four sequences and that were at least 300 bp long) included 3,757 loci and 2,175,243 bp of sequence data; 12.9% of the alignments overall were represented by missing data. The mean locus length was 579 bp (range: 300–1,864). All Illumina reads and UCE sequences are available as NCBI BioProject PRJNA901984 (Suppl. material 1). UCE data were not recovered from the *S.ater* holotype. However, the concatenated ML phylogeny places the Sarawak specimens as sister to *P.muscorum*. The *S.ater + P.muscorum* clade is not aligned with any species in the genus *Suncus* (Fig. 3A). The two-step species tree recovered nearly the same topology as the concatenated ML phylogeny with no changes to the relationship between *S.ater*, *P.muscorum*, and other members of the genus *Suncus* (Fig. 3B).

### ﻿Nomenclature

**Figure 4.**
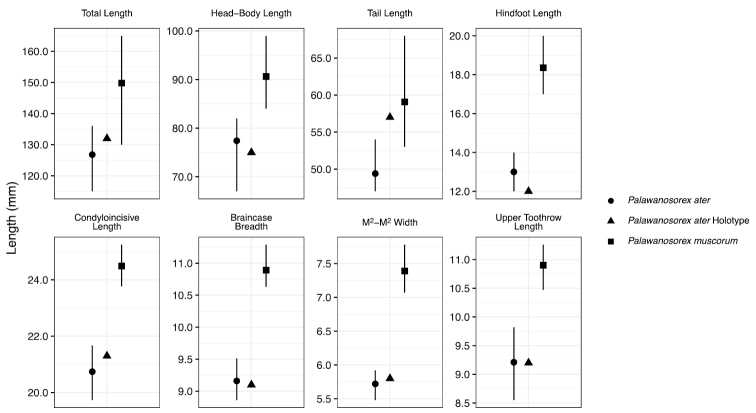
The holotype of *Palawanosorexater* largely matches the external and cranial measurements of *P.ater* specimens captured in Sarawak, not *P.muscorum*. Each panel represents a measurement, and the *y*-axis represents the measurement length in mm. Each species is represented by a different shape. The upper and lower bounds of the point intervals represent the maximum and minimum values for each measurement for each species. Measurements are limited to those reported in the description of *Suncusater* (Medway 1965). *Palawanosorexater* measurements are taken from six specimens collected in Sarawak, Malaysia. *Palawanosorexmuscorum* measurements are taken from Hutterer et al. (2018: tables 1, 2). LSUMZ 40695 has a cropped tail and was removed from the total length and tail length measurements.

**Figure 5.**
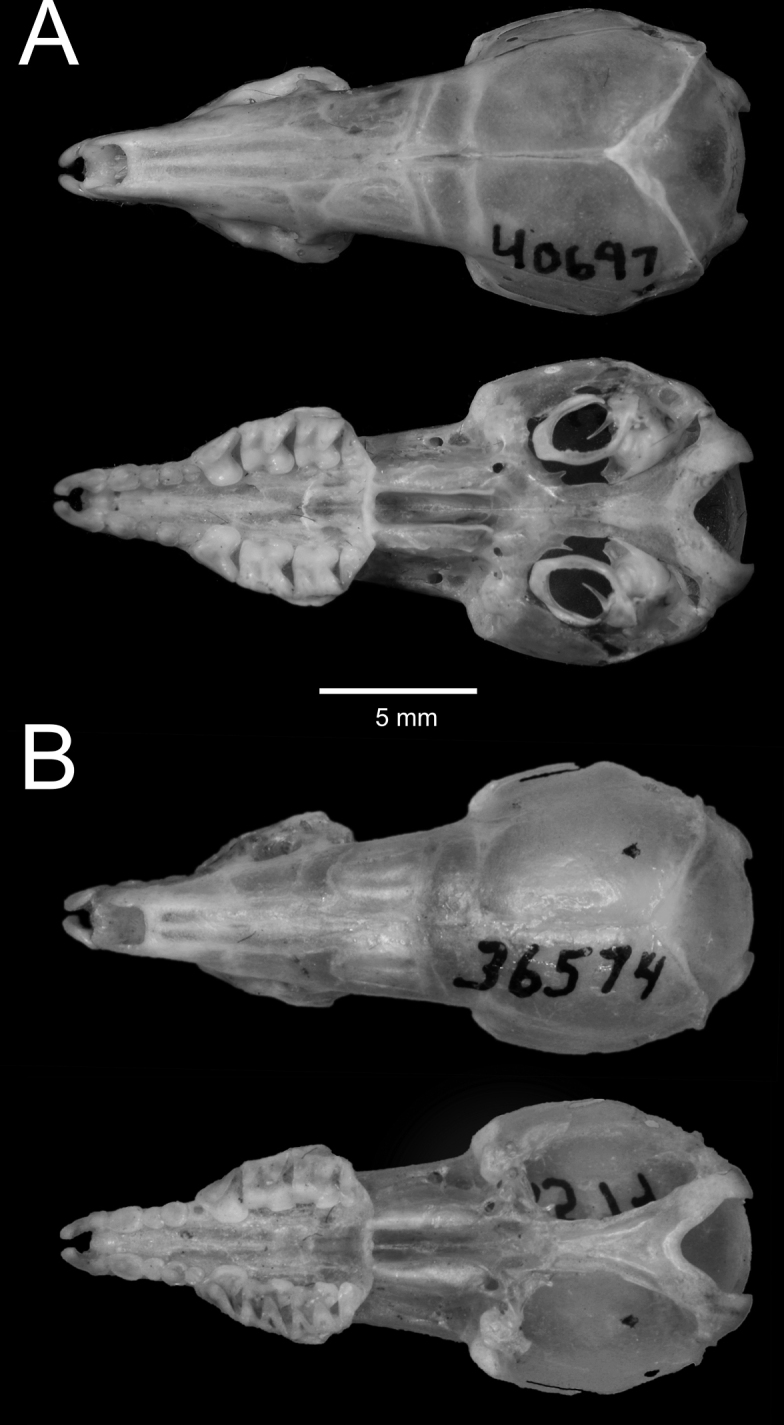
**A** dorsal and ventral views of the cranium of LSUMZ 40697, *Palawanosorexater*, collected on Mount Murud, Sarawak, Malaysia. Photo by Heru Handika **B** dorsal and ventral views of the holotype of *P.ater*, MCZ 36574, collected on Mt. Kinabalu, Sabah, Malaysia. Photo by Museum of Comparative Zoology, Harvard University, President and Fellows of Harvard College.

Mitochondrial DNA from the holotype of *Suncusater* revealed that it is closely aligned with the six newly collected, dark-colored shrews from Borneo, and that this clade is sister to the species *Palawanosorexmuscorum*, a recently described genus and species known only from the Philippine island of Palawan, north of Borneo (Hutterer et al. 2018). Phylogenetic estimates from UCE data support the mitochondrial results, showing that the newly collected Bornean shrews, which are mitochondrially aligned to the *S.ater* holotype, are sister to *P.muscorum*. However, *P.muscorum* is much larger, has a much longer tail that lacks any bristles, and has several cranial characters that clearly distinguish it from *S.ater* (Fig. 4; see detailed comparison by Hutterer et al. 2018: 526), and the average CYTB distance between these two species is 11.77%, indicating a long history of reproductive isolation. Therefore, molecular evidence strongly demonstrates that *S.ater* has a sister relationship with *P.muscorum*. For this reason, we transfer *S.ater* to *Palawanosorex*. Furthermore, based on mitochondrial DNA and morphological data, we also assign the specimens collected in two localities in Sarawak to *P.ater*, substantially increasing the known geographic range of this species and demonstrating that it is not confined to the slopes of Mount Kinabalu in Sabah, Malaysia. We note that many of the proposed synapomorphies of the genus *Palawanosorex* no longer apply. The body size and appendage lengths of *P.ater* are all much smaller than *P.muscorum*. Additionally, as noted by Hutterer et al. (2018), *P.ater* does not have the long claws, bare interdigital surfaces, bristle-free tail, wide antorbital bridge, nor the reduced-size P4 and molars of *P.muscorum*. We do note that, although lacking in the *P.ater* holotype, the Sarawak specimens do have dorsal foramina in various stages of fusion (Fig. 5), similar to *P.muscorum* (Hutterer et al. 2018).

## ﻿Discussion

Our investigation of the phylogenetic placement of the enigmatic Bornean Black Shrew revealed that it represents the second member of the newly described crocidurine genus *Palawanosorex* (Hutterer et al. 2018). As such, five species from three crocidurine genera (*Crocidura*, *Palawanosorex*, and *Suncus*) are considered endemic to Borneo, although recent phylogeographic studies have shown that this number may be an underestimate (Hinckley et al. 2022). All of the native, non-volant mammals endemic to Palawan have their closest relatives in Borneo (Heaney 1986; Esselstyn et al. 2004; Piper et al. 2011). Hutterer et al. (2018) hypothesized, correctly, that, given additional sampling, a close relative of *P.muscorum* would be found on Borneo. These authors anticipated that any newly discovered relatives would share more morphological characters with the type species, which caused the relationship between *P.ater* and *P.muscorum* to go unrecognized (Fig. 4; Hutterer et al. 2018). Generating DNA sequence data from the holotype of *P.ater* was critical in resolving this taxonomic enigma, an approach increasingly used to resolve species limits for poorly known lineages (Kirchman et al. 2010; Kirwan et al. 2015; McGuire et al. 2018; Krabbe et al. 2020; Esselstyn et al. 2021) and groups with convoluted taxonomic histories (Chomicki and Renner 2015; Hedin et al. 2018; Contreras‐Ortiz et al. 2019; Giarla and Voss 2020; Cong et al. 2021; Vences et al. 2022). We also verified the identity of the first modern specimens of *P.ater* following a sampling gap of 80 years since the collection of, and 55 years since the description of, the type of this species.

*Palawanosorexater* was first placed in the genus *Suncus* largely by the presence of the fifth unicuspid. However, Medway (1965) also noted that this species does not resemble any other Southeast Asian crocidurine and suggested that it may be closely aligned with *Suncusdayi* from southern India, which our results refute (Fig. 2). If, prior to the availability of molecular data, simply possessing a fifth unicuspid and therefore not being a *Crocidura* was sufficient evidence to place a species in the genus *Suncus*, then a thorough systematic evaluation of South and Southeast Asian *Suncus* species is warranted. Several *Suncus* species remain poorly studied. The Bornean Pygmy Shrew, *Suncushosei*, has often been aligned with the widespread *Suncusetruscus* complex, though authors have shown some hesitancy in this placement (e.g., Hutterer 2005) and this hypothesis lacks genetic evidence. Similarly, the scantly studied Flores Shrew, *Suncusmertensi* Kock, 1974, was hypothesized to be a relic of an earlier insular fauna (van der Hoek Ostende et al. 2006), the precise pattern that the genus *Palawanosorex* appears to represent (Hutterer et al. 2018). Only through comprehensive surveys of Southeast Asian fauna and targeted sampling of genetic material from historical specimens can we continue to piece together the complex biogeographic history of this dynamic region.

## Supplementary materials

Supplementary material 1 GenBank Accession numbers for genetic sequences used in this study Data type genetic https://binary.pensoft.net/file/785758This dataset is made available under the Open Database License (http://opendatacommons.org/licenses/odbl/1.0/). The Open Database License (ODbL) is a license agreement intended to allow users to freely share, modify, and use this Dataset while maintaining this same freedom for others, provided that the original source and author(s) are credited.Nations JA

Supplementary material 2 Best-fitting nucleotide substitution model for each gene in the mitochondrial DNA analysis Data type genetic https://binary.pensoft.net/file/785759This dataset is made available under the Open Database License (http://opendatacommons.org/licenses/odbl/1.0/). The Open Database License (ODbL) is a license agreement intended to allow users to freely share, modify, and use this Dataset while maintaining this same freedom for others, provided that the original source and author(s) are credited.Nations JA

Supplementary material 3 Maximum-likelihood crocidurine mitochondrial gene tree, without CYTB, inferred in IQTree Data type image (eps file) https://binary.pensoft.net/file/785760This dataset is made available under the Open Database License (http://opendatacommons.org/licenses/odbl/1.0/). The Open Database License (ODbL) is a license agreement intended to allow users to freely share, modify, and use this Dataset while maintaining this same freedom for others, provided that the original source and author(s) are credited.Nations JA
