# Supplementary material for: ﻿Molecular data from the holotype of the enigmatic Bornean Black Shrew, Suncusater Medway, 1965 (Soricidae, Crocidurinae), place it in the genus Palawanosorex
Source: Zookeys. 2022 Dec 21;1137:17–31. doi: 10.3897/zookeys.1137.94217 (PMC9836656; doi:10.3897/zookeys.1137.94217)
Supplement: Supplementary material 2 — Best-fitting nucleotide substitution model for each gene in the mitochondrial DNA analysis [file zookeys-1137-017_article-94217__-s002.docx]

**Table S2:**. ModelFinder results for the 10-gene dataset. For protein-coding genes, codon position is indicated in parentheses after the gene name.

| **Partition Number** | **Best Fitting Model** | **Data Subsets Included** |
| --- | --- | --- |
| 1 | GTR+F+G4 | 16s rRNA, ATP8 (1), ATP8 (2), ND4 (1), ND5 (1) |
| 2 | TIM2e+I+G4 | ATP6 (1), COX1 (1), COX2 (1), COX3 (1), CYTB (1), ND6 (1) |
| 3 | HKY+F+I+G4 | ATP6 (2), COX1 (2), COX2 (2), COX3 (2), CYTB (2), ND6 (2) |
| 4 | TN+F+R3 | ATP6 (3), ATP8 (3), COX1 (3), COX2 (3), COX3 (3), ND4 (3), ND5 (3) |
| 5 | HKY+F+G4 | CYTB (3) |
| 6 | TPM3+F+I+G4 | ND4 (2), ND5 (2) |
| 7 | HKY+F+G4 | ND6 (3) |
